# Traditional Chinese medicine prescription Guizhi Fuling Pills in the treatment of endometriosis

**DOI:** 10.7150/ijms.55789

**Published:** 2021-04-16

**Authors:** Xin Wang, Yaxin Shi, Li Xu, Zilu Wang, Yanfei Wang, Wei Shi, Ke Ma

**Affiliations:** 1 College of Traditional Chinese Medicine, Shandong University of Traditional Chinese Medicine, Jinan 250355, PR China.; 2 Department of Gynecology, Affiliated Hospital of Shandong University of Traditional Chinese Medicine, Jinan 250014, PR China.; 3 Provincial Hospital Affiliated to Shandong First Medical University, Jinan 250012, PR China.

**Keywords:** Endometriosis, *Zheng Jia*, Guizhi Fuling Pills, traditional Chinese medicine, pharmacological

## Abstract

Endometriosis (EMs) is recorded as *Zheng Jia* in traditional Chinese medicine (TCM) books. Guizhi Fuling Pills (GFPs), a classic prescription for promoting blood circulation and removing blood stasis, is widely used for women's blood stasis diseases represented by* Zheng Jia*. At present, it has been applied to treat EMs in clinical settings. In this review, we systematically summarized the active ingredients and pharmacological mechanism of five Chinese herbs contained in GFPs and clinical applications of GFPs. The potential pathways of GFPs in the treatment of EMs were explored through network pharmacology. The current researches results indicate that the mechanisms of GFPs in the treatment of EMs mainly include acesodyne, anti-inflammation and improvement of hemodynamics. The main active compounds that are responsible for pharmacological effects in five Chinese herbs are paeonol, pachymic acid, cinnamaldehyde, amygdaloside and Paeoniflorin. This review can lay the foundation and identify the research direction for the development of GFPs as a new drug therapy for the treatment of EMs.

## Introduction

Endometriosis (EMs) is defined as the presence and growth of endometrial-like tissue outside the uterus, which is mainly characterized by dysmenorrhea, dyspareunia, pelvic pain and infertility 1, 2. It is estimated that the prevalence of EMs is 6-10% and the majority of suffer are women aged 25-35 years old 1, 3. Unfortunately, the understanding of its pathogenesis is insufficient and explicit. It is possibly connected with retrograde menstruation phenomenon, estrogen dependence, progesterone resistance and inflammation 4, 5. Hormonal therapy and surgical therapy are currently common therapy for EMs. However, hormone compounds could only temporarily inhibit the secretion of endogenous ovarian hormones 6, 7. In addition, long-term use of hormones may lead to impaired ovarian reserve function, interference with ovulation, abnormal uterine bleeding and infertility 6, 7. A five-year follow-up study showed that without drug-assisted treatment, the recurrence rate of patients treated by surgery was as high as 40-50% 8. Therefore, it is particularly important to seek new drugs that can treat endometriosis or relieve pain without interfering with ovulation.

TCM pills are one of the main forms of traditional Chinese medicine preparations, which are mostly applied in the clinical treatment of chronic diseases and regulation of body function 9. For example, Liuwei Dihuang Pills was used for curing kidney deficiency as well as Lizhong Pills with the functions of warming and strengthening spleen 9. The gynecology of TCM has a history of 3,000 years. In the book *Synopsis of the Golden Chamber* written by Zhang Zhongjing in the Eastern Han Dynasty, the prevention and treatment of women's pregnancy diseases, postpartum diseases and other common diseases had been systematically recorded. Moreover, the book summarized a wealth of treatment experience and medication principles on gynecology diseases. Classic famous prescription GFPs is a representative TCM pill in this book. Its efficacy is to promote blood circulation, remove blood stasis, eliminate abdominal mass and stop vaginal bleeding. Recently, it has been mainly used in the clinical treatment of EMs as well as adenomyosis (AM) 10, 11. In order to understand the pharmacological mechanism and clinical application of GFPs in the treatment of EMs, we reviewed and summarized the previous clinical and animal studies concerning the treatment of EMs with GFPs, with the aim of clarifying the advantages of TCM in the treatment of gynecological diseases.

## Endometriosis and *Zheng jia*

There is no relevant record about EMs in TCM books. According to its clinical manifestations, it can be considered as *Zheng Jia* in TCM, which was first recorded in *Huangdi Neijing*. Chinese medicine scholars named all the lumps in the human body as *Zheng Jia* 12. *Synopsis of the Golden Chamber* had discussed in detail that lower abdominal pain, abdominal mass and irregular vaginal bleeding were the main clinical manifestations of *Zheng Jia*. Thus, *Zheng Jia*, as a gynecological disease, usually refers to the agglomeration in female lower abdomen accompanied by abdominal distension, pain, vaginal bleeding and other symptoms. AM, EMs, polycystic ovarian syndrome (PCOS) and reproductive system tumors all can be regarded as *Zheng Jia* according to its symptoms and pathological signs 13. Further, the pathogenesis of *Zheng Jia* is also similar to the EMs in TCM. TCM theory explains that the pathogenesis of *Zheng Jia* is mainly related to blood accumulation and qi stagnation in the uterus, which form lumps over time. TCM proposes that EMs is caused by retrograde menstruation, which results in blood stasis blocking the uterus for a long time. Thus, blood stasis is the core pathogenesis and pathological essence of both *Zheng Jia* and EMs.

Based on the pathogenesis of *Zheng Jia*, the relevant therapeutic methods are suggested in the *Synopsis of the Golden Chamber “Chapter 20, the pluse, syndrome and treatment of women during pregnancy”*. Promoting blood circulation, removing blood stasis, warming and dredging meridians are the core therapeutic methods. Subsequently, the classic prescription GFPs is established. The prescription is composed of 5 traditional Chinese herbs: Cassia Twig (*Cinnamomum cassia Presl*), Poria Cocos (*Poria cocos(Schw.)Wolf*), Peach Kernel (*Prunus persica (L.) Batsch*), Red Peony Root (*Paeonia veitchii Lynch*) and Cortex Moutan (*Paeonia suffruticosa Andr.*). Poria Cocos plays a role in infiltrating dampness and strengthening spleen. Peach Kernel and Red Paeony root have effects on promoting blood circulation and relieveing pain. Cortex Moutan is commonly used to clearing the heat caused by blood stasis 14. The combination of the five herbs can maximize the functions in the treatment of EMs 15.

## Pharmacological study on active components of five Chinese herbs of GFPs

### Cassia Twig (CT)

CT which is the dried twig of Cinnamon was first recorded in the *Shen Nong's herbal Classic* and was commonly used in clinic. CT has the efficacy of sweating and slackening muscles, warming and dredging meridians, and assisting yang and transforming qi 16. CT contains a lot of different ingredients, such as benzaldehyde, trans-cinnamaldehyde, organic acids, tannins, sugars, steroids and other components. While the most important ingredient in the volatile oil of CT is cinnamaldehyde 17, 18. Pharmacological research demonstrated that the volatile oil of CT played a positive role in diminishing inflammation, relieving pain, promoting blood circulation and resisting platelet aggregation 17, 19. The occurrence of EMs was associated with the increase of cyclooxygenase-2 (COX-2), vascular endothelial growth factor (VEGF) and tumor necrosis factor alpha (TNF-α) contents as well as changes in NF-κB signal pathway. Chao *el*. indicated that cinnamaldehyde could reduce inflammatory reaction by activating NF-κB signal pathway and reducing the production of interleukin-1 beta (IL-1β) and TNF-α 20. Yao *el.* confirmed that volatile oil of CT could reduce the expression of VEGF, COX-2 and other factors in ovarian tissue of rats, which could effectively treat infertility 21. Furthermore, in the clinical treatment of EMs, CT is often used in combination with other traditional Chinese herbs instead of single application. Studies proved that the compatibility of CT with poria cocos could increase the dissolution rate of cinnamaldehyde by nearly 3 times, which could enhance the efficacy of CT in promoting blood circulation and dredging collaterals 22, 23. Cooperating with peach kernel, the anticoagulant effect is stronger than that of CT alone 24. In conclusion, CT can be applied to treat EMs based on its anti-inflammatory and anticoagulant effects.

### Poria Cocos (PC)

PC is the dry sclerotium of Polyporaceae 25. In TCM clinic, PC which has the effects of eliminating dampness, invigorating spleen, calming heart and tranquilizing mind is applied in many settings 26. Pharmacological researches demonstrated that PC had an active impact on againsting cancer, regulating immune function, diminishing inflammation and preventing oxidization. Furthermore, report found that triterpenes and polysaccharides were the main active components in PK that exerted pharmacological effects 27. Among polysaccharides, pachyman is one of the most important active ingredients, which can regulate immune function, resist inflammation and inhibit tumor. The mechanism of pachyman regulating immune function might be depend on enhancing Natural killer (NK) cell activity and regulating the levels of inflammatory cytokines such as interleukin-2 (IL-2) and TNF-α 28. Besides, triterpenes of PC also plays a crucial role in anti- inflammatory effect. There were evidences to support that it could inhibit the expression of IL-1β through MAPK, PI3K/Akt and NF-κB signaling pathways 29. Research indicated that it was also related to bidirectional regulation of MAPK signaling pathways to inhibit macrophage inflammatory factor expression 30. Moreover, it could induce the release of prostaglandin I2 (PGI2) by upregulation of COX-2 in the MAPK signaling pathway to maintain vascular homeostasis 31. Additionally, pachymic acid could reduce the excessive secretion of nitric oxide, endothelin -1 (ET-1) and thromboxane A2 to inhibit platelet aggregation 32. Meanwhile, EMs involved with multiple signal transduction pathways, such as MAPK, NF-κB, and was closely associated with inflammatory response 33. Lee *el*. proposed that p38MAPK which was phosphorylated by IL-1 was expressed in both ectopic and eutopic endometrium and stimulated inflammatory cells to secrete more interleukin-6 (IL-6), interleukin-8 (IL-8), COX-2 and other inflammatory mediators in EMs patients 34. Khan *el*. suggested that lipopolysaccharide (LPS) stimulated the production of high levels of TNF-α, IL-6 and IL-8 through activating the NF-κB pathway in the ectopenic endometrium stromal cells 35. Therefore, PC can be applied to treat EMs based on its anti-inflammatory and immune function regulating effects.

### Peach Kernel (PK)

PK is the dried mature seed of *Rosaceae* plant peach or mountain peach, which was first recorded in *Shen Nong's Herbal Classic*. The main efficacy of PC includes promoting blood circulation, removing blood stasis, relaxing bowels as well as relieving cough and asthma 36. In TCM clinical setting, it is often used for the treatment of amenorrhea, dysmenorrhea, abdominal mass, traumatic injury, intestinal dryness, constipation, cough, asthma and other diseases 37. Pharmacological studies demonstrated that PK mainly contained fat-soluble substances, proteins, sterols, glycosides, flavonoids, phenolic acids and other active ingredients, which had the effects of anticoagulant, antithrombotic, anti-inflammatory, preventing liver fibrosis and enhancing immunity 38. Report from Zhu* el* confirmed that water extract derived from PK could effectively inhibit platelet aggregation and it was much more effective than the amygdalin and the fat oil of PK 39. Glyceryl trioleate contained in the oil of PK had anticoagulant activity. And the coagulation time extension rate of it was 37% 40. Amygdalin, the main chemical component in PK, exerted anti-inflammatory activity by inhibiting the expression of COX-2 and inducible nitric oxide synthase in mouse BV2 microglia induced by LPS 41. Further, it could effectively relieve inflammatory pain, and the mechanism of action was connected with c-fos and inflammatory factors 42. Thus, PK is beneficial to the treatment of EMs, owning to the functions of pain and inflammation relieving.

### Red Peony Root (RPR)

The medicinal part of *Paeonia lactiflora* is rhizome, which is divided into RPR and *Radix Paeoniae Alba* (RPA). TCM theory consider that RPA is beneficial to nourish yin and tonify deficiency, while RPR is suitable for promoting blood circulation, removing blood stasis and relieving pain 43. Because *Zheng Jia* is blood stasis syndrome, the Paeonia lactiflora in GFPs should refer to RPR. Pharmacological researches indicated that the main components of RPA and RPR were monoterpene, glycoside, tannin and organic acids 44. Although the chemical compositions of RPA and RPR are similar, they are not completely the same. The content of paeony glycoside in RPR was much higher than that of RPA, however, RPA contained more paeony lactone glycoside 44, 45. Report supported that paeony glycoside had significant analgesic effect, and its analgesic effect was better than that of paeony lactone glycoside 46. There was evidence showed that excessive prostaglandin released from ectopic endometrium tissue cause myometrial hypertonus and secondary ischemia, resulting in dysmenorrhea in patients 47, 48. Meanwhile, research demonstrated that paeony glycoside effectively reduced the twisting times of mouse pain model caused by acetic acid induced writhing, and the analgesic mechanism might be related to increasing the level of β-endorphin in cerebral cortex and serum and reducing the secretion of prostaglandin estradiol (E2) 46. Apart from this, study proved that paeony glycoside could improve the blood stasis state of rats with acute blood stasis syndrome by reducing the whole blood viscosity and regulating the balance of ET-1 and NO, and its effect was stronger than that of paeony lactone glycoside 49. Thus, RPR can be applied to treat EMs based on its analgesic and blood circulation promoting effects.

### Cortex Moutan (CM)

CM was first published in *Shen Nong's Herbal Classic*. Its medical effectiveness is clearing heat and cooling blood, promoting blood circulation and removing blood stasis 50. CM contained complex chemical components. The paeonol, paeony glycoside and benzoyl paeoniflorin were the main active compounds 51. Paeony glycoside could alleviate pain obviously 46. Besides, paeonol significantly inhibited the proliferation and differentiation of tumor cells. Study confirmed that the anti-tumor mechanism of paeonol was based on its inhibition of expression of cyclooxygenase -2 (COX-2) gene 52. Correspondingly, inflammation is a typical feature of EMs, and the contents of COX-2, NF-κ B and other cytokines significantly increased in ectopic endometrium 53-55. Consequently, the anti-inflammatory effect of CM is suitable for EMs. In addition, research proposed that paeonol reduced inflammatory response by decreasing the content of IL-1 and IL-6 in the serum of EMs rat model and improve blood viscosity 56.Moreover, evidence supported that benzoyl paeoniflorin could play an anti-inflammatory role by inhibiting COX-1 and COX-2 activities 57.

## Clinical application of GFPs in the treatment of EMs

In the clinical treatment of EMs, GFPs is often changed into decoction, and the proportions of CT, PC, PK, RPR, CM is equal and the usual dose of five Chinese herbs in the prescription is 12 g 58. The Chinese herbs were decocted into 500 ml liquid medicine, and take it in the morning and evening. Besides, for convenience of taking medicine, the herbs were also encapsulated for administration at 0.31 g per capsule. The method of administration is to take three capsules three times a day 59.

In recent years, a series of studies proved that treating EMs with GFPs alone or in combination with western medicine had achieved satisfactory curative effect and was considered as an alternative drug for treating EMs 60-62. GFPs, taken three pills at a time, three times a day, combined with mordern medicine could significantly reduce the levels of Leptin, VEGF and IL-8 in serum of patients with EMs and improve ovarian function. In addition, it has obvious effect on inhibiting the growth of cystic mass and relieving dysmenorrhea 63. Zhang *el.* 64, 65 conducted a randomized controlled trial to compare the clinical efficacy of mifepristone and GFPs combined with mifepristone in the treatment of EMs. The test data indicated that GFPs combined with mifepristone had a better reduction in the levels of CA125, CA199, VEGF, superoxide dismutase (SOD), IL-6, IL-8, TNF-α, hs-CRP than mifepristone alone. The clinical effective rate of the test group was 94.33%, significantly higher than that of the control group 75.47%. Moreover, GFPs has long-lasting effect and low recurrence rate in the treatment of EMs. A 3-month continuous treatment experiment compared the GFPs with the Danazol. The result showed the recurrence rate of GFPs group was 17.4% one year after drug withdrawal, which was lower than 31.8% of the Danazol group 66. Furthermore, GFPs combined with other comprehensive treatment of TCM also applied to treat EMs, for example, TCM enema and acupuncture. Study proposed that the total effective rate of the combination of GFPs and TCM enema for EMs was 96%, which was higher than 80% of the control group 67. In addition, acupuncture aided GFPs can not only get a significant therapeutic effect on EMs but also has fewer side effects. Compared with the control group, the total effective rate is 79.49% and the effective rate for pain relief was 76.92%, which was higher than that of the control group (P<0.05) 68. As a result, under the guidance of syndrome differentiation and treatment theory of TCM, GFPs are appropriated for EMs of blood stasis type 69, 70.

AM is a common gynecologic disease, which is caused by invasion of endometrial glands and stroma into myometrium to form diffuse or localized lesions 71, 72. There are many similarities between AM and EMs in clinical manifestations, pathogenesis and treatment methods. Research demonstrated that GFPs also has good therapeutic effects on AM 73. Relevant studies confirmed that GFPs combined with western medicine was more effective than oral western medicine in the treatment of AM. A randomized clinical trial was designed to compare the clinical effects of GFPs and gestrinone. Patients in control group were given gestrinone orally, 2.5 mg/time, three times a week. Observation group were treated with GFPs on the basis of the control group. The specific dosage and usage of the drug are as follows: CT, CM 12 g each, PK 10 g, RPR 8 g, and PC 9 g, taken orally twice in the morning and evening. The specific dosage and usage of the drug are as follows: CT, CM 12 g each, PK 10 g, RPR 8 g, and PC 9 g, taken orally twice in the morning and evening. The results indicated that the clinical effective rate of the experimental group was 91.7% higher than that of the control group 70.8%, and the levels of IL-6, PGE-2 and ET were lower than those of the control group 74. In addition, the clinical efficacy of radiofrequency ablation plus GFPs was significantly effective than that of radiofrequency ablation alone, and had the advantages of reducing recurrence rate and ameliorating clinical symptoms 75.

## Prediction of potential mechanism of GFPs in treating EMs

TCM has the characteristics of multi-component, multi-target and multi-pathway, as a result, it is difficult to analyze the complex mechanism of TCM only by traditional experimental methods 76. Network pharmacology, as a powerful method integrating system biology, bioinformatics and multiple pharmacology is a reliable approach to explore the potential therapeutic mechanism of herbs 77. It clarifies the complex interactions among genes, proteins and compounds related to diseases and drugs at the network level.

Based on the principle of network pharmacology, we explored the mechanism of GFPs therapy for EMs. Firstly, we confirmed 102 targets of GFPSs from websites TCMSP (http://tcmspw.com/tcmsp.php) and SymMap (http://www.symmap.org/). Second, 304 endometriosis-related genes were obtained from databases DisGeNET (http://www.disgenet.org/) and GeneCards (https://www.genecards.org/). Subsequently, 26 intersection genes were obtained by Venn diagram. Kyoto Encyclopedia of Genes and Genomes (KEGG) analysis was explored to speculate the potential pathway of GFPs treatment for EM. Table 1 presents the 9 concerned pathways. The results of network pharmacology research supported that the potential mechanism of GFPs in the treatment of EMs might be inflammatory pathways, such as, TNF signaling pathway (p<0.01) and NF-kappa B signaling pathway (p<0.01).

## Conclusion and Prospect

Treatment of EMs is a challenge in the field of gynecology. The accompanying dysmenorrhea and infertility have troubled women of childbearing age. However, the current treatment approaches have the defects of side-effect and high recurrence rate. Therefore, it is essential to develop drugs that can target the pathogenesis of EMs and effectively relieves the symptoms of patients without affecting ovulation and pregnancy functions. TCM is a precious resource, and many Chinese herbal medicines are worth exploring. Many clinical practices have proved the advantages of TCM in the treatment of disease, and have gradually attracted worldwide attention. EMs syndrome differentiation belongs to the category of blood stasis syndrome in TCM. GFPs has been used for the treatment of EMs in recent years as a famous classical Chinese medicine prescription commonly applied for the treatment of women's blood stasis disease in history. In order to deeply understand the effective components, mechanism and advantages of GFPs in clinical treatment of EMs, the articles concerned with pharmacology, animal experiments and clinical trials are classified and sorted out. At present, the main functions of medicine monomer in GFPs were focused on anti-tumor, anti-inflammatory, analgesic and hemodynamic improvement. The network pharmacology research also indicated that the potential mechanism of GFPs in the treatment of EMs might be inflammatory pathways, such as, TNF signaling pathway and NF-kappa B signaling pathway. However, it did not fully clarify the mechanism of GFPs as EMs therapeutic drug. For example, the understanding of medicine composition is insufficient. The related researches on medicine ingredients are few and lack pertinence to EMs. The clinical control experiment cases are few. Currently, more and more attention is being paid to the multidisciplinary integration research of TCM. For example, on the basis of classic famous prescriptions of TCM and combined with modern pharmacological research and evidence-based medicine, innovative addition and subtraction of Chinese herbs were carried out. The clinical efficacy of Sanjie Zhentong Capsule which is established by clinical experiences had been proved to be effective in the treatment of EMs 78. Studies proved that the active ingredients Verticinone and Verticine in Sanjie Zhentong Capsule had significant analgesic and anti-inflammatory activities 79. Besides, Apigenin had estrogen-like effect on animals, and it inhibited ovarian secretion of estrogen and progesterone 80. Thus, multidisciplinary integration will all help promote the modernization of TCM and the development of gynecology of TCM.

## Figures and Tables

**Figure 1:**
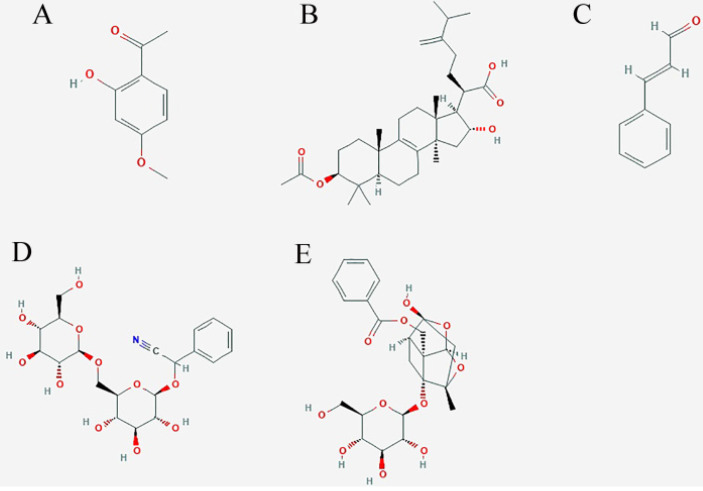
Structural formula of five important compounds in five Chinese herbs of GFPs. A: paeonol; B: pachymic acid; C:cinnamaldehyde; D: amygdaloside; E: Paeoniflorin.

**Figure 2:**
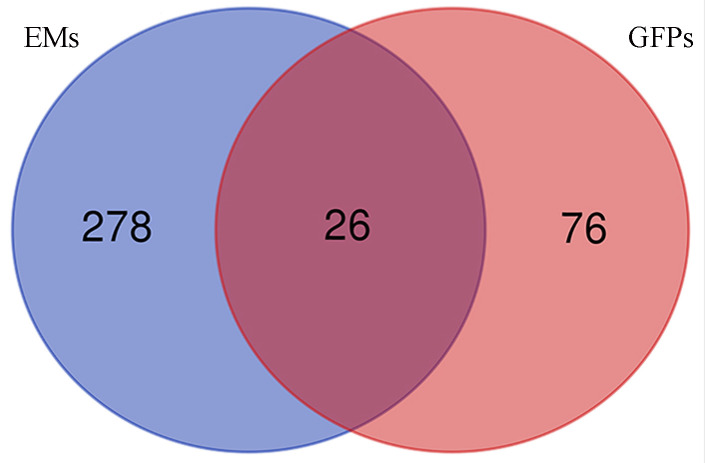
The intersection genes of GFPs and Ems.

**Figure 3:**
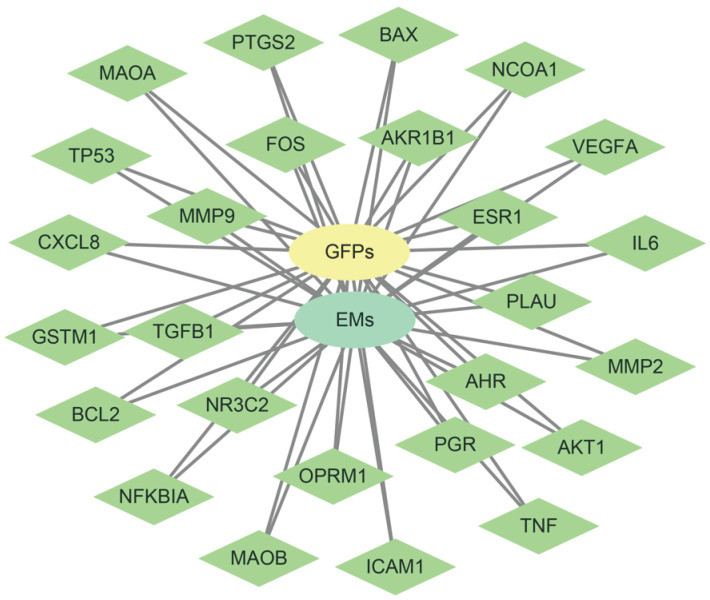
** 26 disease targets have established networks with GFPs and EMs.** Green node represents intersection gene, yellow node represents GFPs, and blue nod represents EMs.

**Table 1:** The information of 11 potential target pathways

ID	Term	Count	P-value
hsa05200	Pathways in cancer	13	1.48E-09
hsa04668	TNF signaling pathway	8	5.03E-08
hsa05205	Proteoglycans in cancer	9	2.19E-07
hsa04064	NF-kappa B signaling pathway	7	3.86E-07
hsa04210	Apoptosis	6	1.88E-06
hsa04915	Estrogen signaling pathway	6	1.91E-05
hsa05202	Transcriptional misregulation in cancer	5	0.002435561
hsa05206	MicroRNAs in cancer	6	0.002657271
hsa04066	HIF-1 signaling pathway	4	0.004310234

**Table 2:** The TCM efficacy and main components of five Chinese herbs in GFPs

Chinese herbs	TCM efficacy	Main components
Cassia Twig	Sweating and slackening muscles, warming and dredging meridians, and assisting yang, transforming qi	Benzaldehyde, trans-cinnamaldehyde, organic acids, tannins, sugars, steroids
Poria Cocos	Eliminating dampness, invigorating spleen, calming heart, tranquilizing mind	Triterpenes, polysaccharides
Peach Kernel	promoting blood circulation, removing blood stasis, relaxing bowels , relieving cough and asthma	fat-soluble substances, proteins, sterols, glycosides, flavonoids, phenolic acids
Red Peony Root	Promoting blood circulation, removing blood stasis, relieving pain	Monoterpene, glycoside, tannin,organic acids
Cortex Moutan	Clearing heat, cooling blood, promoting blood circulation, removing blood stasis	Paeonol, paeony glycoside and benzoyl paeoniflorin

## Abbreviations

EMs: endometriosis
TCM: traditional Chinese medicine
GFPs: Guizhi Fuling pills
AM: adenomyosis
PCOS: polycystic ovarian syndrome
CT: Cassia Twig
PC: Poria Cocos
PK: Peach Kernel
RPR: Red Peony Root
RPA: Radix Paeoniae Alba
CM: Cortex Moutan
LPS: lipopolysaccharide
COX-2: cyclooxygenase-2
VEGF: vascular endothelial growth factor
TNF-α: tumor necrosis factor alpha
IL-1β: interleukin-1 beta
NK: natural killer
IL-2: interleukin-2
PGI2: prostaglandin I2
IL-6: interleukin-6
IL-8: interleukin-8
ET-1: endothelin-1
E2: estradiol
SOD: superoxide dismutase
KEGG: Kyoto Encyclopedia of Genes and Genomes
